# Complement component 3 deficiency prolongs MHC-II disparate skin allograft survival by increasing the CD4^+^ CD25^+^ regulatory T cells population

**DOI:** 10.1038/srep33489

**Published:** 2016-09-19

**Authors:** Quan-you Zheng, Shen-ju Liang, Gui-qing Li, Yan-bo Lv, You Li, Ming Tang, Kun Zhang, Gui-lian Xu, Ke-qin Zhang

**Affiliations:** 1 Department of Nephrology, Southwest Hospital, Third Military Medical University, Chongqing 400038, China; 2 Department of Urology, Daping Hospital, Third Military Medical University, Chongqing 400042, China; 3 Department of Rheumatism and Immunology, Daping Hospital, Third Military Medical University, Chongqing 400042, China; 4 Department of Immunology, Third Military Medical University, Chongqing 400038, China

## Abstract

Recent reports suggest that complement system contributes to allograft rejection. However, its underlying mechanism is poorly understood. Herein, we investigate the role of complement component 3 (C3) in a single MHC-II molecule mismatched murine model of allograft rejection using C3 deficient mice (C3^−/−^) as skin graft donors or recipients. Compared with C3^+/+^ B6 allografts, C3^−/−^ B6 grafts dramatically prolonged survival in MHC-II molecule mismatched H-2^bm12^ B6 recipients, indicating that C3 plays a critical role in allograft rejection. Compared with C3^+/+^ allografts, both Th17 cell infiltration and Th1/Th17 associated cytokine mRNA levels were clearly reduced in C3^−/−^ allografts. Moreover, C3^−/−^ allografts caused attenuated Th1/Th17 responses, but increased CD4^+^CD25^+^Foxp3^+^ regulatory T (Treg) cell expression markedly in local intragraft and H-2^bm12^ recipients. Depletion of Treg cells by anti-CD25 monoclonal antibody (mAb) negated the survival advantages conferred by C3 deficiency. Our results indicate for the first time that C3 deficiency can prolong MHC-II molecule mismatched skin allograft survival, which is further confirmed to be associated with increased CD4^+^ CD25^+^ Treg cell population expansion and attenuated Th1/Th17 response.

The complement system is one of the major contributors to innate immunity and contains a series of soluble and cell surface proteins, including plasma components, specific receptors and diverse regulators. It serves as the first line of defense against invading pathogens by applying complement activation elements acting with antibodies, phagocytes or through the formation of the membrane attack complex (MAC)^1^,^2^. In addition to its vital role in innate immunity, increasing evidence has indicated that the complement system regulates adaptive immunity, especially the antigen-specific T cell response^3^,^4^. Furthermore, the complement system plays a critical role in transplantation success, and has been shown to participate in the pathogenesis of ischemia-reperfusion injury, to promote alloantibody-mediated rejection, to modulate the alloreactive T cell response and to contribute to progressive chronic allograft rejection^5^,^6^,^7^.

Three pathways of complement activation: the classic pathway, lectin pathway, and alternative pathway, which of them converge at the level of complement component 3 (C3) convertase, from which functional products are generated in a sequential manner^5^. Studies have demonstrated that C3 plays a critical role in the regulation of T cell responses to autoimmune disease, viral infection and transplant rejection^8^,^9^,^10^,^11^,^12^. As compared with systemic C3 produced in the liver, local C3 is mainly generated by diverse tissue-resident cells (such as tubular cells in the kidney, endothelial and epithelial cells), antigen presentation cells (APCs) and T cells^13^,^14^,^15^,^16^,^17^. The role of C3 in allograft rejection remains controversial. It was indicated that intra-renal C3 deficiency (C3^−/−^) prolonged renal allograft survival and caused a defective alloreactive T cell response when compared with C3 positive (C3^+/+^) allografts^18^. On the contrary, in a minor H disparate skin transplant model, results suggested that C1q or C3 deficiency (C1q^−/−^ or C3^−/−^) accelerated graft rejection as well as impaired intranasal tolerance induction^19^.

Several studies have revealed that C3 deficiency is associated with attenuated Th1/Th17 responses^14^,^20^,^21^. CD4^+^ CD25^+^ Foxp3^+^ regulatory T (Treg) cells are thought to be critical for promoting peripheral tolerance, limiting chronic inflammatory disease and relieving autoimmune disease^22^,^23^,^24^. Strikingly, the absence of C3aR and C5aR signalling in CD4^+^ T cells has recently been confirmed to favour Treg expansion and survival^25^,^26^. Nevertheless, it remains unclear how C3 affects Treg cells development and regulates the balance between Treg cells and Th1/Th17 cells response during transplantation rejection.

In this study, we investigated the role of C3 in a single MHC-II molecule mismatched murine model of allograft rejection. We demonstrate that graft C3 deficiency can clearly prolong skin allograft survival, partly by expanding the population of CD4^+^ CD25^+^ Treg cells and attenuating Th1/Th17 cell responses in recipients with C3^−/−^ allografts.

## Results

### Increased survival of C3^−/−^ allografts in an MHC-II molecule disparate skin transplant model

**Figure 1:**
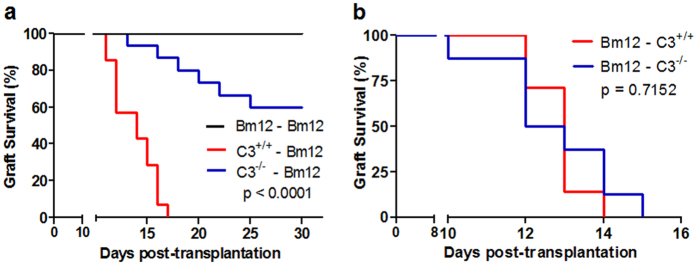
Local C3 deficiency prolonged MHC-II disparate skin allograft survival. Mice were transplanted with full thickness tail skin from indicated donors, and transplant rejection was monitored. (**a**) Alloreactive C3^+/+^ B6 or C3^−/−^ B6 skin grafts were transplanted into MHC-II disparate Bm12 recipient mice. Syngeneic Bm12 donors were used as negative controls (n = 6–15). (**b**) Bm12 mice skin grafts were transplanted into C3^+/+^ or C3^−/−^ B6 recipient mice (n = 7–8). Data were obtained from two independent experiments.

To explore the exact role of C3 in allograft rejection, we established an MHC-II molecule disparate skin transplant model. Full thickness tail skin from alloreactive C3^+/+^ B6 and C3^−/−^ B6 mice was transplanted into MHC-II molecule disparate Bm12 recipients, and syngeneic Bm12 used as a negative control. Compared with C3^+/+^ allografts, which were completely rejected within 17 days after transplantation, 60% of C3^−/−^ allografts survived until day 30 (Fig. 1a, p < 0.0001). These results indicate that C3 deficiency could prolong MHC-II disparate skin allograft survival. It was previously reported that the T cells in C3^−/−^ mice have no intrinsic defect^27^,^28^. Consistent with these results by Carroll *et al.*, we found that, for Bm12 allografts, no difference in survival was observed in C3^+/+^ and C3^−/−^ recipient mice (median survival 13.5 days vs. 14.0 days, p = 0.7152, Fig. 1b).

### Decreased inflammatory cell infiltration in C3^−/−^ allografts

**Figure 2:**
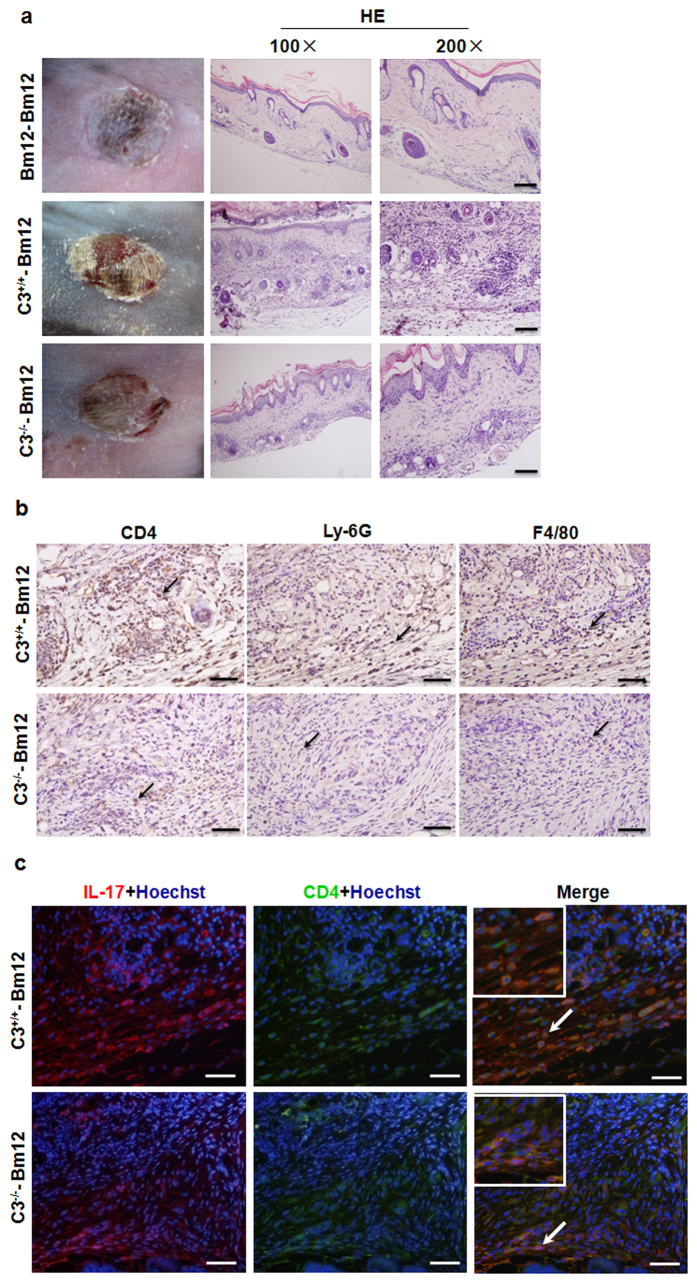
Decreased inflammatory cell infiltration in C3^−/−^ allografts. Bm12 mice were transplanted with C3^+/+^, C3^−/−^ or Bm12 grafts. Rejection monitored from day 7 and following experiments performed at day 10 after transplantation. (**a**) Macroscopic aspects of skin grafts and histology of graft sections stained with haematoxylin and eosin. (**b**) Skin allografts were stained for CD4, Ly-6G and F4/80. Magnification: ×400. (**c**) Th17 cell infiltration in allografts was determined by immunofluorescence. Grafts sections were stained with anti-CD4 antibody (green), anti-IL-17 antibody (red), and Hoechst 33258 for nucleus (blue). Magnification: ×400. Clearly positive stained cells were pointed out with arrows (Scale bar: 5 μm).

In contrast to the extensive necrosis and large-scale mononuclear cell infiltration found in C3^+/+^ allografts on day 10 post-transplantation, C3^−/−^ allografts were relatively intact and only displayed a small amount of mononuclear cell infiltration (Fig. 2a). Furthermore, immunohistochemistry results showed that CD4^+^ T, neutrophil (Ly-6G) and macrophage (F4/80) infiltration in C3^−/−^ allografts was remarkably lower than that of C3^+/+^ allografts (Fig. 2b). Th17 (CD4^+^ IL-17^+^ T) cells, which are believed to play a vital role in allograft rejection, were further analysed. As showed in Fig. 2c, compared with the C3^+/+^ allografts, Th17 cells in C3^−/−^ allografts were noticeably decreased. These results demonstrate that the prolonged survival of C3^−/−^ grafts may be associated with reduced inflammatory cell infiltration.

### C3 deficiency impaired inflammatory cytokines and chemokines expression

**Figure 3:**
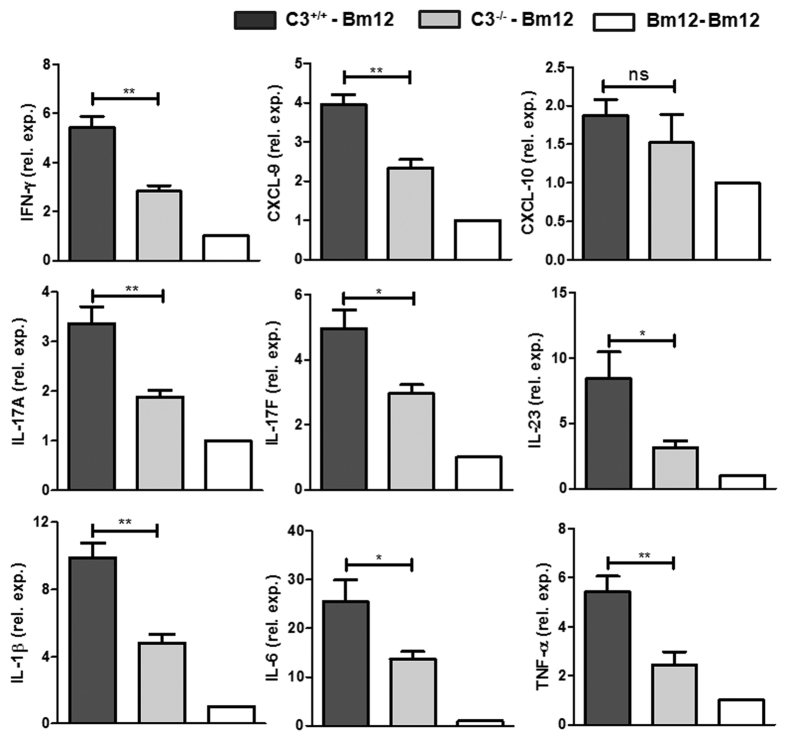
C3 deficiency led to reduced inflammatory cytokine and chemotactic factor expression in allografts. Bm12 mice were transplanted with tail skin grafts from Bm12 (Isografts), C3^+/+^ and C3^−/−^ (allografts) mice. Grafts were harvested on day 10 after transplantation and expression of inflammatory cytokine and chemokine mRNA levels in grafts were measured by RT-qPCR (n = 5–6). One representative data set from three independent experiments is shown. *p < 0.05; **p < 0.01.

Considering the important role of cytokines and chemokines in inflammatory cell infiltration, their expression was investigated. Compared with C3^+/+^ allografts, C3^−/−^ allografts displayed remarkably attenuated the mRNA levels of critical cytokines (IFN-γ, IL-17 and IL-23) and chemokine CXCL-9, which are associated with Th1 and Th17 effective T (Teff) cell responses (Fig. 3). C3^−/−^ allografts also displayed reduced mRNA levels of IL-1β, IL-6, TNF- α (Fig. 3). These results suggest that the absence of C3 in allografts leads to impaired expression of Teff cytokines.

### C3^−/−^ allografts induced weak lymphocyte proliferation and Th1/Th17 response in Bm12 recipients

**Figure 4:**
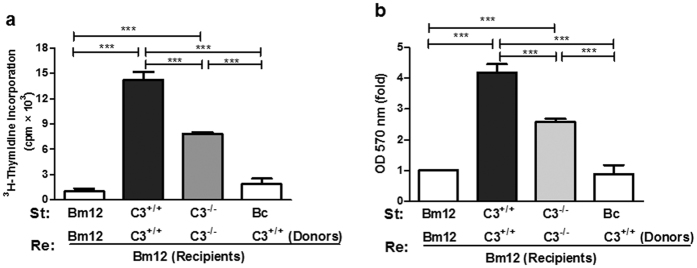
C3^−/−^ allografts induced impaired T cell proliferation in Bm12 recipient mice. Bm12 mice were transplanted with tail skin grafts from Bm12, C3^+/+^ and C3^−/−^ mice, respectively. Draining lymph node cells (axilla) were collected on day 10 after transplantation and used as the response cells (Re.). The irradiated (2000 rad) naïve splenocytes from Bm12, C3^+/+^, C3^−/−^ and third-party Bc were used as stimulation cells (St.). The Re. cells were co-cultured with St. cells in 96 well-plates for 72 hours. The cells in culture received 0.5 uCi ^3^H-thymidine in the last 16 hours and ^3^H incorporation was determined by liquid scintillation counting (**a**). T cell proliferation was also measured by MTT assay (**b**). The data are presented as mean ± SD and are representative of three independent experiments. ***p < 0.001.

**Figure 5:**
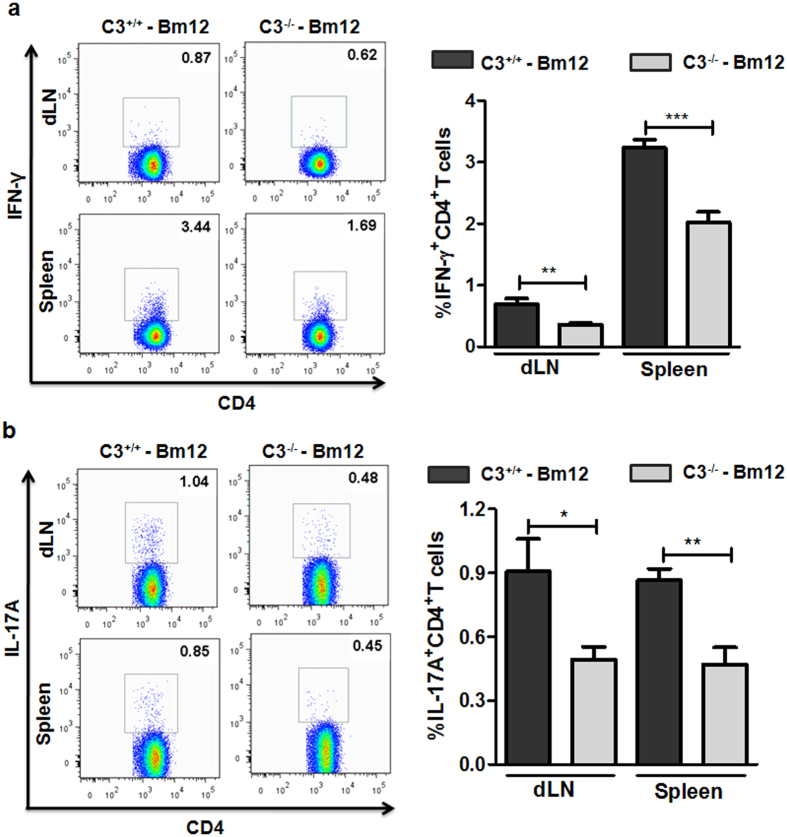
C3^−/−^ allograft induced attenuated Th1 and Th17 responses in Bm12 recipient mice. Bm12 mice were transplanted with tail skin allografts from C3^+/+^ and C3^−/−^ mice, respectively. Splenocytes and draining lymph node cells (axilla) of Bm12 recipient mice were prepared on day 14 after transplantation, and Th1 (IFN-γ^+^ CD4^+^ T) and Th17 (IL-17^+^ CD4^+^ T) responses were assessed by FACS. (**a**) Percentage of IFN-γ^+^ CD4^+^ T cells. (**b**) Percentage of IL-17^+^ CD4^+^ T cells. The data are representative of two individual experiments. *p < 0.05; **p < 0.01; ***p < 0.001.

To further explore the underlying mechanism that accounted for increased C3^−/−^ allograft survival, the response of lymphocytes in Bm12 recipients including the baseline of CD4^+^IFN-γ^+^ T and CD4^+^IL-17^+^ T cells in naïve Bm12 mice (supplementary Figure 1a,b) was assessed. It was found that lymphocyte proliferation (Fig. 4a,b) and the percentage of CD4^+^ IFN-γ^+^ (Th1)/CD4^+^ IL-17^+^ (Th17) cells (Fig. 5a,b) in recipients with C3^−/−^ allografts was significantly reduced compared with C3^+/+^ allografts. These results revealed that specific alloantigen lymphocyte priming and Th1/Th17 response was impaired in recipients with C3^−/−^ allografts.

### C3^−/−^ allografts caused increased CD4^+^ CD25^+^ Treg cell expression in local intragrafts and recipients

**Figure 6:**
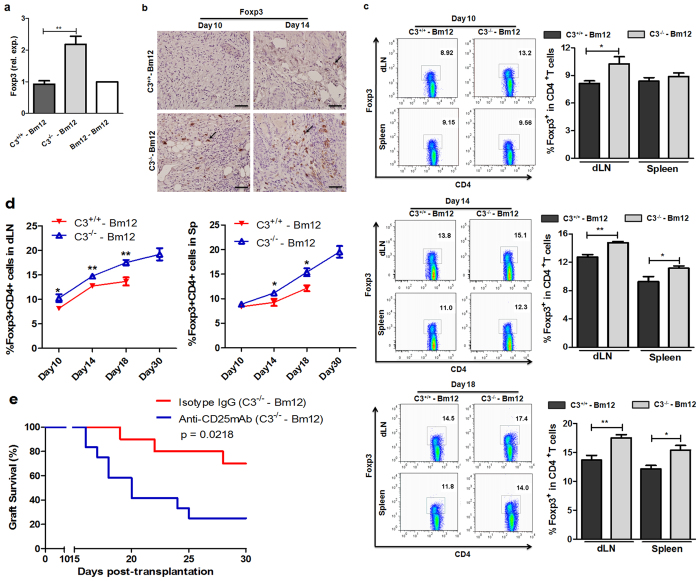
C3^−/−^ allografts led to increased CD4^+^ CD25^+^ Treg cell induction in Bm12 recipient mice. Bm12 mice were transplanted with tail skin grafts from Bm12 (Isografts), C3^+/+^ and C3^−/−^ (allografts) mice. (**a**,**b**) Grafts were harvested at the indicated time points after transplantation. Foxp3 expression in allografts was measured by RT-qPCR on day 10 (**a**) and IHC on day 10 and day 14 (**b**). (**c**,**d**) Splenocytes and draining lymph node cells (axilla) of Bm12 recipients were prepared at the indicated time points after transplantation and the percentage of Foxp3^+^ CD4^+^ T cells was measured by FACS. (**e**) Neutralizing anti-CD25 mAb or isotype control IgG were administrated to Bm12 recipients with C3^−/−^ tail skin allografts at 0 days, 2 days and 2 weeks post-transplantation. (n = 10–12). The data were representative of three individual experiments. Positive stained cells were pointed out with arrows and the scale bar represents a length of 5 μm. *p < 0.05; **p < 0.01.

CD4^+^ CD25^+^ Foxp3^+^ Treg cells play a critical role in graft tolerance induction^22^,^23^,^24^. C3 deficiency could promote CD4^+^ CD25^+^ Treg cell differentiation in STZ-induced diabetic mice^29^. As shown in Fig. 6a,b, a clear increase in Foxp3 expression was observed in C3^−/−^ grafts. Although the percentage of CD4^+^ CD25^+^ Treg cells increased gradually after transplantation in recipients with C3^+/+^ grafts and recipients with C3^−/−^ grafts (supplementary Figure 1c), in the same time point the frequency of CD4^+^ CD25^+^ Treg cells in the latter was significantly higher (Fig. 6c,d). Depletion of CD4^+^ CD25^+^ Treg cells following administration of anti-CD25 mAb, led to a marked decrease in C3^−/−^ graft survival (Fig. 6e), indicating a close correlation between CD4^+^ CD25^+^ Treg cell induction and increased C3^−/−^ graft survival.

### C3^−/−^ DCs have an increased regulatory T cell driving capacity *in vitro*

**Figure 7:**
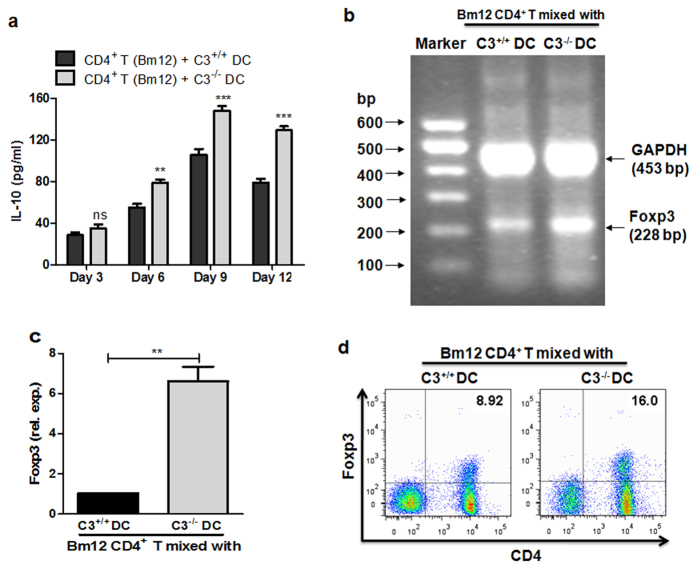
C3^−/−^ DCs have increased regulatory T cell driving capacity *in vitro*. Following stimulation with LPS for 24 h, the BMDC cells (8 × 10^4^) from either naïve C3^+/+^ or C3^−/−^ mice were irradiated, and then co-cultured with naïve alloreactive CD4^+^ T cells (2 × 10^5^) from Bm12 mice for up to 12 days. (**a**) IL-10 levels in supernatants were examined by ELISA. (**b**,**c**) Foxp3 expression was analysed by RT-PCR or qPCR after 9 days co-culture. GAPDH was used as the house keeping gene. The 600-bp DNA markers are shown on the left. (**d**) The protein levels of Foxp3 in these co-cultured CD4^+^ T cells were examined by FACS. One representative result from three individual experiments is shown. **p < 0.01; ***p < 0.001.

It has been demonstrated that the presenting of alloantigen to T cells of the recipient by donor antigen presentation cells (APCs) is a key step during allograft rejection^30^. Dendritic cells (DCs), one of the most important APCs, were able to expand the population of antigen specific Treg cells^31^,^32^. IL-10 is a key cytokine in Treg cell development. After co-culturing C3^+/+^ or C3^−/−^ bone marrow DCs (BMDCs) with naïve alloreactive CD4^+^ T cells, it was found that levels of IL-10 in the supernatant rapidly increased; peaking at 9 days (Fig. 7a). Moreover, the secretion of IL-10 by T cells cultured with C3^−/−^ DCs, was remarkably higher than in T cells cultured with C3^+/+^ DCs (6 days, P < 0.01; 9 days, P < 0.001; 12 days, P < 0.01; (Fig. 7a). As a specific marker for CD4^+^ CD25^+^ Treg cells, the expression of Foxp3 in CD4^+^ T cells initiated by BMDCs was tested by RT-PCR, qPCR, and flow cytometry. It was shown that compared with C3^+/+^ DCs, C3^−/−^ DCs initiated dramatically increased expression of Foxp3 in naïve alloreactive CD4^+^ T cells (Fig. 7b–d). All these results indicate that C3^−/−^ DCs possess higher regulatory T cell driving capacity than that found in C3^+/+^ DCs, in this MHC-II disparate skin graft model, which may contribute to the increased induction of Treg cells in recipients with C3^−/−^ allografts.

## Discussion

Several reports have demonstrated that complement C3 plays a critical role in transplantation progress^8^,^9^,^10^,^11^,^12^. Local activation of C3 enhanced renal ischemia reperfusion damage, promoted renal graft rejection and increased alloreactive T cell responses^18^,^33^. In a clinical study, it was shown that the expression of C3 was up-regulated in both humoral and cellular renal graft rejection, with epithelial tubular cells being a major source of C3^34^. Jeffrey *et al.* suggested that local intra-renal complement C3 production following donor brain death is closely associated with renal graft function early after transplantation^35^. Consistent with these studies, our results show that local complement C3 deficiency prolongs MHC-II molecular disparate skin graft survival, whilst the deficiency of systemic complement C3 in recipients could not extend skin graft survival. Therefore, abrogation of complement expression in the donor allograft is a promising therapeutic approach for extending skin graft survival.

In contrast to the deteriorative role of complement C3 mentioned above, it was found that the deficiency of C3 accelerates the rejection of minor H mismatched skin grafts and that C3 is required to facilitate HY-peptide mediated tolerance by modulating DC function^19^. The discrepancy between these results may be related to the distinct immune responses initiated by different graft antigens. The minor HY peptide induced a syngeneic immune response. On the other hand, grafts in the MHC-II molecule disparate skin graft model caused the alloreactive immune response, which more closely matched clinical models.

C3 is a pivotal regulator in alloreactive T cell responses^5^,^11^,^14^,^18^. C3 mediates Th1/Th17 polarization and blocking C3 signalling reduces Th1/Th17 responses^20^,^21^. CD4^+^ CD25^+^ Treg cells are thought to be critical for promoting peripheral tolerance^22^,^23^,^24^,^36^. It was previously reported that primary C3 deficiency, which was defined as an autosomal disease, was associated with impaired Treg cell population expansion, capacity after stimulation of CD4^+^ T cells with IL-2, CD3 and CD46^37^. Accumulating studies have demonstrated that both Th17 and Treg cells are reciprocal to each other^38^,^39^,^40^. Consistent with these results, we found enhanced Foxp3 expression and attenuated Th1/Th17 responses in C3^−/−^ allografts. Depletion of CD4^+^ CD25^+^ Treg cells, following treatment with anti- CD25 mAb, increased the Th17 cells percentage (supplementary Figure 2) in Bm12 recipients and abrogated the survival advantage in C3^−/−^ allografts. This indicates that Treg cells associates the attenuated Th1/Th17 responses and tolerance of C3^−/−^ allografts, at least in this MHC class II molecule disparate skin graft model.

Donor APCs play an important role in the initiation and maintenance of allograft rejection^5^,^41^. DCs are the most potent APCs involved in the priming of T cell responses^42^. The study by Peng *et al.* indicated that C3 synthesized by DCs is required for full CD4^+^ T cell responses^14^, but the underlying mechanism was not fully elucidated. In agreement with Peng’s findings, it was showed that both the DCs numbers and costimulatory molecule CD80 expression of DCs were obviously reduced in C3^−/−^ allografts (supplementary Figure 3), which might be related to the attenuated Th1/Th17 responses. In according, we found that by culturing donor DCs with recipient naïve CD4^+^ T cells, C3-deficient DCs more successfully promoted Treg cell expansion by *in vitro* culturing donor DCs with recipient naïve CD4^+^ T cells, in this MHC class II molecule disparate skin graft model. It is unclear at this point, whether the attenuated Th1/Th17 responses are directly affected by a defect in DC in C3^−/−^ allografts or it occurs indirectly by the C3^−/−^ DC-induced increased Treg cells, or both. This needs to be addressed in the future.

In the current study, we chose a single MHC-II molecule disparate skin graft as the model due to the following reasons: first, the efficiency of local or systemic complement C3 could be easily assessed by utilizing this model, allowing us to further investigate the underlying mechanism. In other models, notably the full MHC mismatched skin transplant model, allografts were rejected rapidly making it hard to perform further studies; Second, by preventing CD8^+^ T cell alloresponses, this MHC-II-CD4^+^ T cell restricted transplant model allowed us to specifically explore the reciprocal interaction among CD4^+^ T cell subsets (such as Th1, Th17 and Treg cells). Indeed, studies have shown that MHC-І-CD8^+^ T cell restricted responses led to the earlier production of IFN-γ, which has a potent regulatory effect on CD4^+^ T cell immune activation^39^,^40^; Finally, the complement C3 produced by APCs was proven to be critical for antigen presentation (including alloantigen) through an MHC class II molecule dependent pathway^43^.

All together, we demonstrate that C3 deficiency extends MHC-II molecule disparate skin allograft survival via promotion of CD4^+^ CD25^+^ Treg cell population expansion and attenuating Th1/Th17 response in recipients. This involves increased Treg driving capacity of DCs in C3^−/−^ allografts. Our data indicate that targeting the complement component C3 of intra-donor organs could plausibly be developed as a clinical approach for improving allograft tolerance.

## Methods

### Mice

C57BL/6.C-H-2^bm12^ (H-2^bm12^ or Bm12) mice and C3^−/−^ C57BL/6 mice (B6 background) were purchased from Jackson Laboratory (Bar Harbor, ME, USA). C3^+/+^ C57BL/6 (B6) and BALB/c (Bc) mice were obtained from the Animal Institute of the Academy of Medical Science (Beijing, China). Animals were bred in a specific pathological free facility. Female mice at 8–12 weeks old were used in all experiments. The using mouse in this study was approved by the Institutional Animal Care and Use Committee of Third Military Medical University. That all experimental protocols were carried out in “accordance” with the approved guidelines.

### Skin Transplantation

Skin grafting was performed using an adapted version of the Billingham and Medawar protocols^44^. Briefly, mice were anaesthetized with 1% pentobarbital sodium in 0.9% normal saline solution according to body weight. A total of 100 μg/kg body weight was administered by intra peritoneal injection (i.p). Full-thickness tail skin (0.8–1.0 cm^2^) obtained from donors was transplanted into the lateral dorsal thoracic wall of recipients. The graft site was covered with Vaseline gauze and an adhesive bandage (BAND∙AID) for 7 days. Thereafter, skin graft survival was assessed daily, and tissue deemed rejected when less than 10% viable tissue remained.

### Histology

For assessing leukocyte infiltration into skin allografts, recipient mice were sacrificed by cervical dislocation and samples retrieved, fixed in 4% paraformaldehyde for 24 hours, embedded in paraffin, and 4 μm sections obtained. The sections were deparaffinised and rehydrated using decreasing concentrations of ethanol, before using for immunohistochemistry (IHC) or immunofluorescence (IF) applications. Antigen retrieval was performed in 10 mM sodium citrate solution for 15 minutes in a microwave oven. For blocking the internal peroxidase, slides were immersed in 3% H_2_O_2_/PBS solution for 15 minutes, washed three times with PBS, and incubated with 5% bovine serum albumin/PBS for 60 minutes. Primary antibodies against CD4, Ly-6G, F4/80, Foxp3, and IL-17 from Santa Cruz (Dallas, Texas, USA) and antibodies for mouse CD11c and CD80 (eBioscience, San Diego, CA, USA) were diluted according to the protocols provided and incubated at 4 °C overnight. Dylight^TM^ 488 donkey anti-rabbit IgG and Cy3^TM^ goat anti-mouse IgG purchased from BioLegend (San Diego, CA, USA), or horse radish peroxidase (HRP) labelled secondary antibodies purchased from Beyotime (Shanghai, China), were incubated for 60 minutes at room temperature. For IHC, after washing with PBS, the 3,3′-diaminobenzodine (Beyotime, Shanghai, China) chromogen solution was added and incubated for 2 to 5 minutes at room temperature, after which slides were counterstained with haematoxylin and then dehydrated. For IF, nuclei were stained with Hoechst 33258 solution (Sigma-Aldrich, St. Louis, MO, USA) at 5 μg/ml, followed by washing with PBS. All sections were examined by light or fluorescence microscopy (Olympus, Tokyo, Japan).

### Mixed lymphocyte response

T cell alloreactive priming capacities were assessed by the standard mixed lymphocyte response. Draining lymph node cell suspension was prepared from Bm12 recipients of C3^+/+^ or C3^−/−^ skin grafts at 10 days after transplantation. After washing twice with PBS, cells at 3 × 10^6^ cells/ml were supplied with complete medium (RPMI 1640 medium containing 10% fetal calf serum, 100 μg/ml streptomycin, 100 U/ml penicillin, 2 μM glutamine, 50 μM β-mercaptoethanol) then 100 μl aliquots were added to a round bottomed 96-well plate. Irradiated splenocytes were collected from syngeneic Bm12, C3^+/+^, C3^−/−^ and third-party Bc mice, then 100 μl aliquots of 3 × 10^6^ cells/ml were added into the indicated wells. After 56 hours, each well of cultured cells was pulsed with 0.5 uCi ^3^H-thymidine for 16 hours. ^3^H incorporation was determined by liquid scintillation counting. Additionally, T cell proliferation was also examined by MTT assay (Sigma-Aldrich, St. Louis, MO, USA) as previously described^45^.

### Conventional RT-PCR

Total RNA was obtained from skin grafts or cell pellets using TRIzol reagent (Biomed, Beijing, China). cDNA was synthesized by applying a PrimeScript^TM^ RT reagent kit (Takara, Shiga, Japan). PCR was performed with 2 μl cDNA and 0.5 μM of primer pairs either for Foxp3 or GAPDH genes, in 20 μl of mixed reaction buffer (Novoprotein, Shanghai, China). Each of the 35 proliferation cycles was divided as follows: 1 min at 94 °C, 1 min at 59 °C, and 1 min at 72 °C. PCR products were visualized following electrophoresis on 2% agarose gel with Golden View staining (Beyotime, Shanghai, China).

### Quantitative RT-PCR

Real-time PCR was conducted with an MxPro3000P (Agilent Stratagene, Santa Clara, CA, USA) and a SYBR^®^ Premix Ex Taq^TM^ reagent kit (Takara, Shiga, Japan). PCR was performed with 2 μl cDNA and 0.5 μM of primer pairs for testing genes of interest or the GAPDH control (Table 1), in 10 μl of mixed reaction buffer. Amplification tests were performed according to the manufacturer’s protocols and applied in triplicate. Levels of gene expression were calculated utilizing the ΔΔCt method^46^. Samples were normalized to the expression level of GAPDH. Primer pair sequences are shown in Table 1.

### Flow Cytometry

Cell suspensions were collected from the graft-draining lymph nodes and spleens of graft recipients. Levels of intracellular cytokines were determined after cells were stimulated with 50 ng/ml pyromellitic acid (PMA), 500 ng/ml ionomycin (Sigma-Aldrich) and 3 μg/ml brefeldin A (BFA) (eBioscience, San Diego, CA, USA) for 4 hours in complete medium. Antibodies against mouse CD3, CD4, IFN-γ (BioLegend, San Diego, CA, USA), Foxp3 and IL-17 (eBioscience, San Diego, CA, USA) were used. Cells at 1 × 10^6^ cells/ml were incubated for 20 min with Fc block and then stained for surface markers (CD3 and CD4) for another 25 min on ice in a dark room. IFN-γ, Foxp3 and IL-17 intracellular staining were performed by utilizing eBioscience Fixation/Permeabilization according to the protocols recommended. The stained cells were assessed using a Canto II flow cytometer (BD, Franklin Lakes, NJ, USA) and data were analysed using Flow Jo software (Tree Star, Oregon, USA).

### CD25^+^ cell depletion

Depletion of CD4^+^ CD25^+^ regulatory T cells was conducted by intra-peritoneal injection (i.p) of 200 μg/mouse anti-CD25 mAbs (PC61) (BD, Franklin Lakes, NJ, USA) on days 0, 2 and 2 weeks post-transplantation. About 90% CD4^+^CD25^+^ cells were depleted, confirmed by flow cytometry (data not shown).

### DC cultures

Bone marrow dendritic cells (BMDCs) were generated from C3^+/+^ or C3^−/−^ mice using a modified protocol of previously described methods^47^. In brief, BM cells were harvested from mouse femurs and tibias. After washing, cells were cultured in 6-well plates at a density of 1 × 10^6^ cells/ml in 2 ml/well DC culture medium (RPMI 1640 medium containing 10% foetal calf serum, 100 μg/ml streptomycin, 100 U/ml penicillin, 2 μM glutamine, 50 μM β-mercaptoethanol, 20 ng/ml granulocyte-macrophage colony-stimulating factor (GM-CSF) (Peprotech, Rocky Hill, NJ, USA). Medium was replaced every other day. At day 7, dislodged cells were collected and then stimulated with 1 μg/ml lipopolysaccharide (LPS) for a further 24 hours to allow the cells to mature. The purity of DCs was routinely no less than 85%, as assessed by flow cytometry.

### Preparation of CD4^+^ T cells

Naïve CD4^+^ T cells were prepared from the spleen of Bm12 mice utilizing an EasySep^TM^ Mouse Naïve CD4^+^ T Cell Isolation Kit (Stem cell Technologies, London, UK). After isolation, the purity of CD4^+^ T cells was routinely more than 90%, as determined by flow cytometry.

### Analysis of alloreactive T cell response

Irradiated (2000 rad) BMDCs (8 × 10^4^/well) from C3^+/+^ or C3^−/−^ mice were co-cultured with purified alloreactive Bm12 naïve CD4^+^ T cells (2 × 10^5^/well) in complete cell culture medium. The supernatant was collected at days 0, 3, 6, 9, 12, and IL-10 levels measured by ELISA (BioLegend, San Diego, CA, USA) according to the protocol provided by the manufacturer. For RNA extraction and Foxp3 expression analysis by flow cytometry, 4 × 10^5^ irradiated DCs and 1 × 10^6^ purified CD4^+^ T cells were co-cultured for 9 days in 48-well plates.

### Statistical analysis

Results are shown as the mean ± standard deviation. GraphPad Prism 5.0 software (La Jolla, CA, USA) was used for statistical analysis. Graft survival was performed graphically by the Kaplan-Meier method and the differences between groups analysed by the log-rank test. A Student’s *t* test or two-way ANOVA was utilized for detecting significant differences among samples, and p < 0.05 was considered statistically significant.

## Additional Information

**How to cite this article**: Zheng, Q.-Y. *et al.* Complement component 3 deficiency prolongs MHC-II disparate skin allograft survival by increasing the CD4^+^ CD25^+^ regulatory T cells population. *Sci. Rep.* **6**, 33489; doi: 10.1038/srep33489 (2016).

## Supplementary Material

Supplementary Information

## Figures and Tables

**Table 1:** Primer pairs sequences of genes interest.

Gene	Primer sequence
IL-6 Forward	5-CCAAGAGGTGAGTGCTTCCC-3
IL-6 Reverse	5-CTGTTGTTCAGACTCTCTCCCT-3
IL-23 Forward	5-ATGCTGGATTGCAGAGCAGTA-3
IL-23 Reverse	5-ACGGGGCACATTATTTTTAGTCT-3
TNF-α Forward	5-TCTTCTCATTCCTGCTTGTGG-3
TNF-α Reverse	5-GGTCTGGGCCATAGAACTGA-3
CXCL 9 Forward	5-TCCTTTTGGGCATCATCTTCC-3
CXCL 9 Reverse	5-TTTGTAGTGGATCGTGCCTCG-3
CXCL10 Forward	5-CCAAGTGCTGCCGTCATTTTC-3
CXCL10 Reverse	5-GGCTCGCAGGGATGATTTCAA-3
IFN-γ Forward	5-ATGAACGCTACACACTGCATC-3
IFN- γ Reverse	5-CCATCCTTTTGCCAGTTCCTC-3
IL-17A Forward	5-TTTAACTCCCTTGGCGCAAAA-3
IL-17A Reverse	5-CTTTCCCTCCGCATTGACAC-3
IL-17F Forward	5-TGCTACTGTTGATGTTGGGAC-3
IL-17F Reverse	5-AATGCCCTGGTTTTGGTTGAA-3
IL-1β Forward	5-GCAACTGTTCCTGAACTCAACT-3
IL-1β Reverse	5-ATCTTTTGGGGTCCGTCAACT-3
Foxp3 Forward	5-ACCTACAGGCCCTTCTCCAG-3
Foxp3 Reverse	5-CTGATCATGGCTGGGTTGTC-3
GAPDH Forward	5-ACCACAGTCCATGCCATCAC-3
GAPDH Reverse	5-TCCACCACCCTGTTGCTGTA-3
