# Supplementary material for: Complement component 3 deficiency prolongs MHC-II disparate skin allograft survival by increasing the CD4+ CD25+ regulatory T cells population
Source: Sci Rep. 2016 Sep 19;6:33489. doi: 10.1038/srep33489 (PMC5027598; doi:10.1038/srep33489)
Supplement: Supplementary Information [file srep33489-s1.pdf]

Complement component 3 deficiency prolongs MHC-Ⅱ disparate skin allograft survival by increasing the CD4$^+$ CD25$^+$ regulatory T cells population

Quan-you Zheng[1, 2], Shen-ju Liang[3], Gui-qing Li[4], Yan-bo Lv[4], You Li[1], Ming Tang[1], Kun Zhang[1], Gui-lian Xu[4], Ke-qin Zhang[1, *]

[1]Department of Nephrology, Southwest Hospital, Third Military Medical University, Chongqing 400038, China.

[2]Department of Urology, Daping Hospital, Third Military Medical University, Chongqing 400042, China.

[3]Department of Rheumatism and Immunology, Daping Hospital, Third Military Medical University, Chongqing 400042, China.

[4]Department of Immunology, Third Military Medical University, Chongqing 400038, China.

* Corresponding Author at: Department of Nephrology, Southwest Hospital, Third Military Medical University, Chongqing 400038, P.R. China. E-mail address: zhkq2004@163.com (K.-Q. Zhang).

Supplementary Figures and Legends:

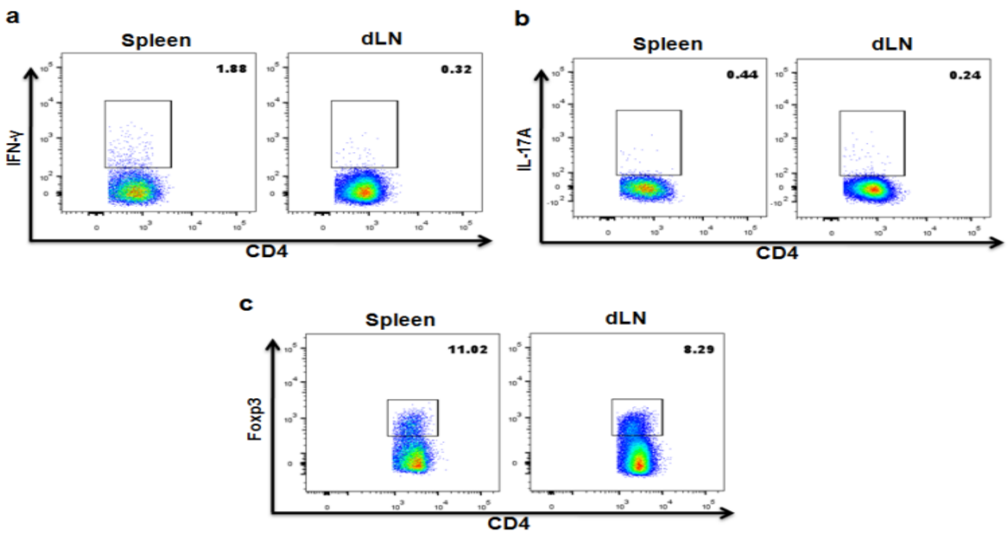

Supplementary Figure 1. Baseline of Th1, Th17, and Treg cells in spleen and dLN of naive Bm12 recipients. Baseline of Th1 (a), Th17 (b), and Treg (c) in spleen and dLN of naive Bm12 mice were assessed by FACS. Representative images from five mice were shown.

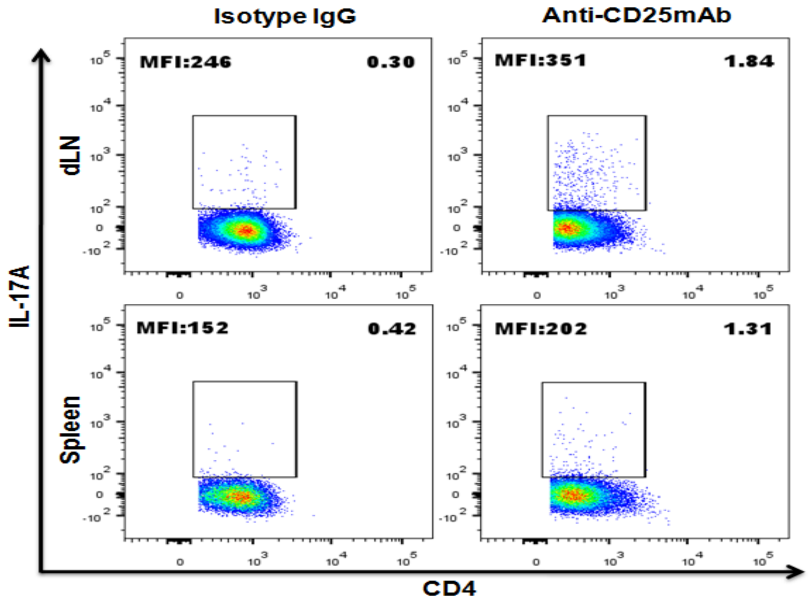

Supplementary Figure 2. Enhanced CD4^{+}IL-17^{+} T cell percentage by depletion of CD25^{+} cells. Depletion of CD25^{+} cells was performed by intra-peritoneal injection

(i.p) of 200 μg/mouse anti-CD25 mAb in Bm12 mice. After 3 days, the percentage of $CD4^{+}IL\text{-}17^{+}$ T cells in spleen and dLN was measured by FACS. Typical images from five mice were shown.

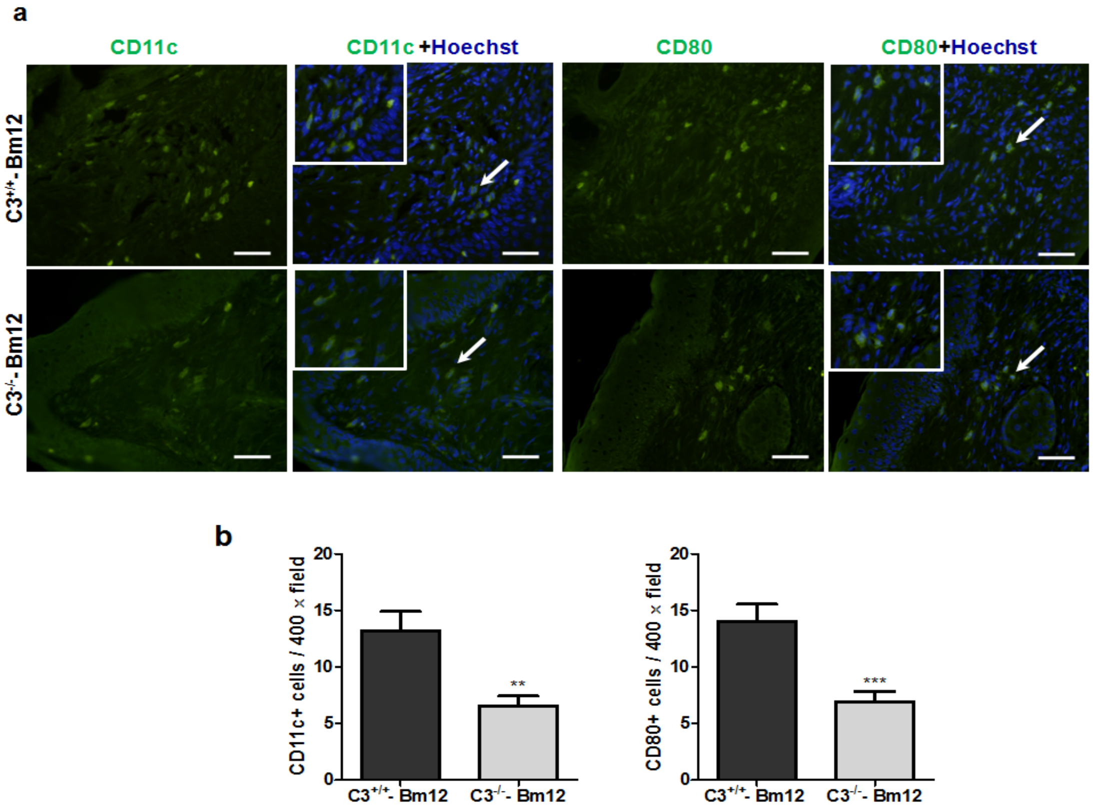

Supplementary Figure 3. Reduced dendritic cells accumulation in $C3^{-/-}$ allografts. Bm12 mice were transplanted with full thickness tail skin allografts from $C3^{+/+}$ and $C3^{-/-}$ mice. (a) Allografts were harvested on day 10 after transplantation and infiltrated dendritic cells were tested by IF. Grafts sections were stained with anti-CD11C antibody (green), anti-CD80 antibody (green), and Hoechst 33258 for nucleus (blue). (b) Dendritic cells infiltration and CD80 expression were analysed by checking the number of positive cells per high-power field (×400). Scale bar in the images represent a length of 5 μm. Clearly positive stained cells were pointed out with white arrows. **, $p < 0.01$; ***, $p < 0.001$.
